# Chemical Profiling of *Jatropha* Tissues under Different Torrefaction Conditions: Application to Biomass Waste Recovery

**DOI:** 10.1371/journal.pone.0106893

**Published:** 2014-09-05

**Authors:** Taiji Watanabe, Amiu Shino, Kinya Akashi, Jun Kikuchi

**Affiliations:** 1 Graduate School of Medical Life Science, Yokohama City University, Tsurumi-ku, Yokohama, Japan; 2 RIKEN Center for Sustainable Resource Science, Tsurumi-ku, Yokohama, Japan; 3 Faculty of Agriculture, Tottori University, Tottori, Japan; 4 Biomass Engineering Program, RIKEN Center for Sustainable Resource Science, Tsurumi-ku, Yokohama, Japan; 5 Graduate School of Bioagricultural Sciences and School of Agricultural Sciences, Nagoya University, Chikusa-ku, Nagoya-shi, Japan; University of Nottingham, United Kingdom

## Abstract

Gradual depletion of the world petroleum reserves and the impact of environmental pollution highlight the importance of developing alternative energy resources such as plant biomass. To address these issues, intensive research has focused on the plant *Jatropha curcas*, which serves as a rich source of biodiesel because of its high seed oil content. However, producing biodiesel from *Jatropha* generates large amounts of biomass waste that are difficult to use. Therefore, the objective of our research was to analyze the effects of different conditions of torrefaction on *Jatropha* biomass. Six different types of *Jatropha* tissues (seed coat, kernel, stem, xylem, bark, and leaf) were torrefied at four different temperature conditions (200°C, 250°C, 300°C, and 350°C), and changes in the metabolite composition of the torrefied products were determined by Fourier transform-infrared spectroscopy and nuclear magnetic resonance analyses. Cellulose was gradually converted to oligosaccharides in the temperature range of 200°C–300°C and completely degraded at 350°C. Hemicellulose residues showed different degradation patterns depending on the tissue, whereas glucuronoxylan efficiently decomposed between 300°C and 350°C. Heat-induced depolymerization of starch to maltodextrin started between 200°C and 250°C, and oligomer sugar structure degradation occurred at higher temperatures. Lignin degraded at each temperature, e.g., syringyl (S) degraded at lower temperatures than guaiacyl (G). Finally, the toxic compound phorbol ester degraded gradually starting at 235°C and efficiently just below 300°C. These results suggest that torrefaction is a feasible treatment for further processing of residual biomass to biorefinery stock or fertilizer.

## Introduction

The gradual depletion of world petroleum reserves together with the impact of environmental pollution by increasing exhaust emission has created an urgent need to develop alternative energy sources [1], [2], [3]. To address these issues, *Jatropha curcas* has been the focus of intensive research, because its seeds contain high levels of oil that is a valuable source of biodiesel. Unfortunately, large amounts of waste are generated by biodiesel production, and these waste include *Jatropha* tissues obtained after harvesting and pruning as well as the seed cake generated by oil extraction. These represent potential valuable resources of carbon for industrial and agricultural use. For example, Gunaseelan reported that the production of *J. curcus* plantation on rain fed dry land at a density of 4444 plants/ha yielded 1.42 ton/ha oil extraction, whereas residual biomass from de-oiled cake, pruned leaves, and fruit hulls yielded 4.83 ton/ha [4]. Furthermore, it was reported that the energy gain from biodiesel exhibited 53 GJ, whereas the energy gain from the anaerobic fermentation of residual biomass exhibited 36 GJ. Therefore, large amounts of residual biomass of *Jatropha* plantation can be used to recycle industrial energy through anaerobic fermentation [5], [6].

Plant lignocellulosic biomass possesses undesirable properties such as high oxygen content, low calorific value, hydrophilicity, and high moisture content [4], [7]. In addition, the chemical composition of plant lignocellulosic biomass is heterogenous, making the design and operation of biorefinery stock production more complicated. Therefore, a key challenge is to develop efficient and cost-effective conversion technologies for maximizing the utilization of lignocellulosic biomass. We have recently reported how ionic liquids can break down strong intermolecular hydrogen bonds in crystalline cellulose [8], [9]. However, at present, this pretreatment technology is rather highly expensive and not environmentally friendly. Furthermore, we have elucidated that anaerobic fermentation sludge can break down crystalline cellulose, and these anaerobic microbiota can immediately produce biogas such as methane [5], [6], [10]. However, these microbiota can immediately metabolize glucose and oligosaccharides; therefore, it is quite difficult to extract biorefinery stock from cellulosic material.

On the other hand, torrefaction pretreatment is a thermal method for the conversion of biomass by heating it from 200°C to 300°C under atmospheric conditions in the absence of oxygen [11], [12], [13], [14]. This process improves biomass properties and was therefore proposed as a potential solution to the problems described above [6], [7], [8], [9]. Torrefied products such as gas, char, and tar (oil) can be used as chemical and energy resources and also as a fertilizer in the form of biochar [15].

Torrefaction has been applied to diverse biomass sources [16], [17], [18], [19]. However, the physicochemical conversion of the plant biomass during torrefaction is largely uncharacterized. Detailed characterization of its chemical composition is required to determine how biomass can be most effectively applied. Therefore, we attempted to determine the physicochemical properties of torrefied *Jatropha* biomass.

**Figure 1:**
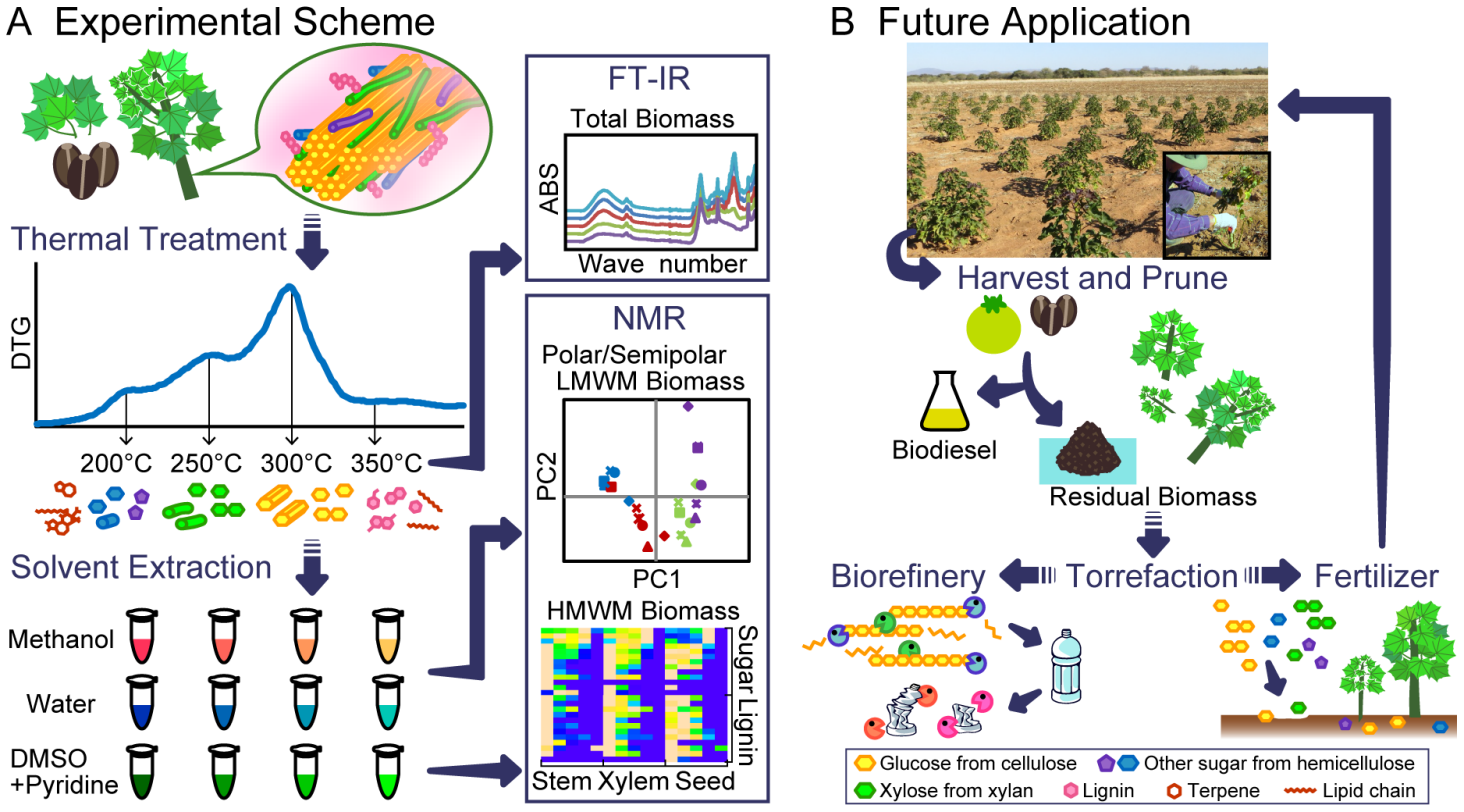
Schematic conceptual figure of the analysis of heat treated *Jatropha* biomass and its future industrial and agricultural application. Torrefaction was applied and compounds present in the biomass were analyzed using FTIR and NMR techniques. FTIR characterized the total residue generated by the treatments, and NMR characterized samples prepared according to their solubilities and molecular weights. These analyses indicate that torrefaction can be more efficiently applied to utilize biomass (A). Scheme of *Jatropha curcas* biodiesel production from farming, pruning, harvesting, and oil extraction. These processes generate biomass from plant tissues and the seed cake (B).

Nuclear magnetic resonance (NMR) is widely used to analyze lignocellulose. The (2D)-NMR heteronuclear single quantum coherence (HSQC) method is useful for characterizing solubilized biomass such plant cell wall and particle organic matter [20], [21], [22], [23], [24], [25], [26]. The objective of the present study was to analyze the products of *Jatropha* biomass conversion using different torrefaction conditions. Torrefied products from different *Jatropha* tissues were analyzed using attenuated total reflectance Fourier transform infrared (ATR-FTIR) and NMR using polar and semipolar low and high molecular weight molecules (LMWMs and HMWMs, respectively. HMWMs refer to the DMSO-solubilized polymer fraction after water and methanol extractions, whereas LMWMs refer to the directly extracted metabolite fractions using water or methanol). Based on these analyses, we aimed to provide information that will guide more efficient application of biomass, such as biorefinery stock and fertilizer (Figure 1).

## Materials and Methods

*Jatropha* stems, seeds, and leaf tissues were harvested as described previously [20]. The stems were analyzed either as a whole or divided into xylem and bark; the latter included the phloem. The seed was divided into coat and kernel; the latter comprised the endosperm and embryo. The samples were freeze-dried and ground using an auto-mill machine (Tokken Co. Ltd, Japan). Powdered samples from the seed coats and kernels were subjected to oil extraction treatment by adding 1 ml of hexane per 100 mg of the sample. Note that the residual oil should be negligible, given that neither FT-IR nor NMR detected corresponding oil signals. The samples were incubated for 5 min at 50°C with shaking, centrifuged at 14000 rpm for 5 min, and then the supernatants discarded. This procedure was repeated five times.

LMWMs were extracted with 100% methanol and then with distilled water. One milliliter of methanol was added to 100 mg of the sample, incubated for 5 min at 50°C with shaking, centrifuged at 15000 rpm for 5 min, and then the supernatant was discarded. This procedure was repeated four times, and the samples were then dried and extracted with Milli-Q water using the same procedure as methanol extraction but without using methanol.

Torrefaction was conducted using an EXSTAR TG/DTA (Thermo Gravimetric/Differential Thermal Analysis) 6300 (SII Nanotechnology Inc., Tokyo, Japan) under a nitrogen atmosphere. The samples were heat-treated at 5°C/min starting at 24°C up to either 200°C, 250°C, 300°C, or 350°C. When the maximum temperature was reached, it was maintained for 10 min.

The torrefied and non-torrefied samples were analyzed in triplicate by ATR-FTIR using a Nicolet 6700 spectrometer (Thermo Fisher Scientific Inc., Waltham, MA, USA) and a KBr disk. The ATR Smart iTR accessory with a high-pressure clamp (Thermo Fisher Scientific Inc., Waltham, MA, USA) was used. Spectra (4500–650 cm^−1^) were obtained using triangular apodization with a resolution of 4 cm^−1^ and an interval of 1 cm^−1^. Background and sample spectra were normalized from 32 scans. The baseline and ATR corrections for penetration depth and frequency variations were conducted using OMNIC software supplied with the equipment.

After FTIR analysis, LMWMs were extracted with 100% methanol and distilled water as described previously [20]; however, the supernatant was collected in the present study. The remaining pellets were freeze-dried and ball-milled for 12 h using a P-5 Ball-mill machine (Fritch, Co. Ltd, Germany) programmed to grind for 10 min at 10 min intervals. The samples were suspended in dimethylsulfoxide (DMSO)–d_6_/pyridine-d_5_ (4∶1) using 60 mg of the sample and 600 µL of solvent. The mixture was incubated at 50°C for 30 min with shaking, centrifuged at 15000 rpm for 5 min, the supernatant collected as HMWMs, and then analyzed using the ^1^H–^13^C-HSQC NMR method. It was performed at 318 K with 32 scans as described previously [27], [28], [29]. The central DMSO solvent cross-peak was used as the internal reference (δC 39.5, δH 2.49 ppm).

LMWM samples collected from 108 mg of torrefied products were dried and dissolved in 600 µL of methanol-d_4_ (CD_3_OD) and deuterium oxide (D_2_O), both containing 1 mM sodium 2,2-dimethyl-2-silapentane-5-sulfonate (DSS). The mixtures were analyzed using the ^1^H-NMR method at 298 K with 64 scans. The DSS signal was used as an internal reference (0.0 ppm). All NMR measurements were obtained using a AvanceII-700 spectrometer (Bruker, MA, USA) equipped with an inverse triple resonance CryoProbe with a Z-axis gradient for 5-mm sample diameters operating at 700. 153 MHz ^1^H frequency. The assignment of the signals in the NMR spectra was performed using SpinAssign (http://prime.psc.riken.jp/) according to previous reports [30], [31], [32], [33], [34], [35], [36].

For the phorbol ester degradation assay, torrefaction of phorbol 12-myristate 13-acetate and phorbol 12,13-dibutyrate (Sigma-Aldrich, St. Louis, MO, USA) as a standard was conducted using an EXSTAR TG/DTA 6300 as described above. The torrefied samples treated at different temperatures were analyzed using ^1^H-NMR at 298 K with 64 scans.

## Results and Discussion

### ATR–FTIR analysis

The treatment temperature was defined through a preliminary thermogravimetric analysis where all the samples were vaporized at a heating rate of 5°C/min from 24°C to 500°C. This analysis showed differential TG peaks at approximately 200°C, 250°C, 300°C, and 350°C (Figure S1).

**Figure 2:**
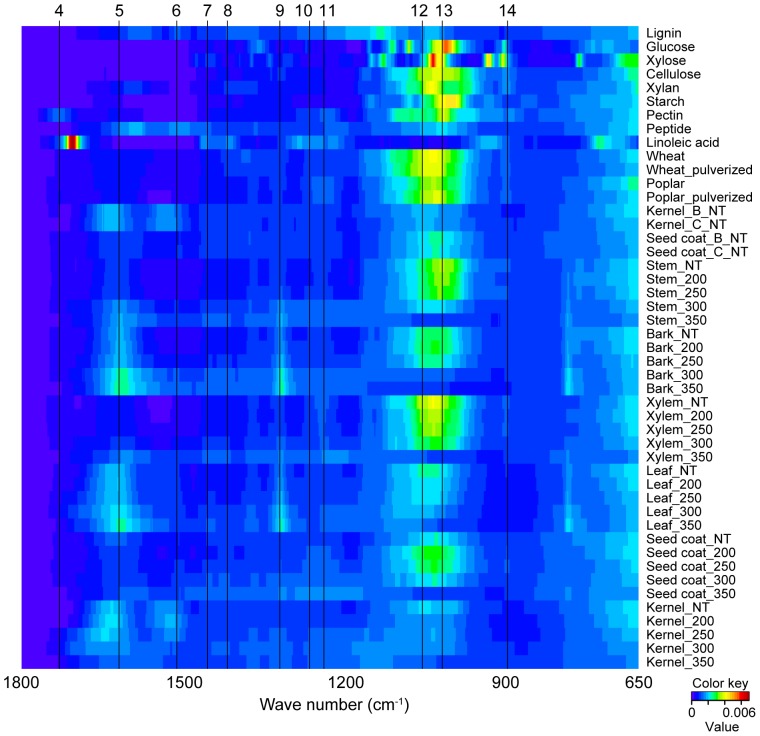
Heat-map like bird's eye viewing of FTIR spectra. Horizontal axis shows wave number and vertical axis shows different samples, and signal intensity (arbitrary unit) are shown according to color key. Top nine samples in vertical axis are standard compounds such as lignin, sugars, peptide and fatty acid. NT, nontreatment. 200, 250, 250, 300 and 350 samples were torrefied at 200°C, 250°C, 300°C, and 350°C, respectively.

To determine the change in chemical composition of *Jatropha* biomass during torrefaction, six different *Jatropha* tissues were treated at four different temperatures (200°C, 250°C, 300°C, and 350°C) and analyzed using ATR–FTIR. Table 1 show the spectra and assignment of functional groups. The peaks were assigned according to the previous reports [20], [23], [37], [38], [39]. Figure 2 shows heat-map like bird's eye viewing of FTIR spectra. The horizontal axis shows wave number and the vertical axis shows different samples, and the signal intensity (arbitrary unit) are shown according to the color key. Top nine samples in the vertical axis are standard compounds such as lignin, sugars, peptide, and fatty acid. The corresponding peaks from low molecular metabolites such as glucose, xylose, and linoleic acid were not present in both non-treated and torrefied biomass spectra. The comparison of three kinds of *Jatropha* varieties of kernel and seed coat did not show any remarkable changes, whereas non-treated stem biomasses from different species (Poplar, Wheat, and *Jatropha*) also did not exhibited remarkable changes. Likewise, mechanical treatment for both Poplar and Kernel samples also did not show remarkable changes. However, heat treatment to *Jatropha* tissues exhibited dramatic changes in FTIR spectra. The intensity of peak number 1 gradually decreased with increased temperature. Some of the observed difference between the 200°C-treated and non-treated samples may represent dehydration of compounds [39], whereas the decrease at higher temperatures may represent the cleavage of intramolecular hydrogen bonds [37].

**Table 1:** Assignment of functional groups in FTIR spectra and the spectra of different samples.

Peak	Wave number (cm^−1^)						Assignment
	seed coat	kernel	stem	bark	xylem	leaf	
1	3288	3257	3298	3294	3309	3298	O-H stretching (hydrogen-bonded)
2	2910	2914	2904	2906	2897	2912	C-H stretching
3	2846	2856	2873	2844	2833	2842	C-H stretching
4	1705	-	1693	1695	1722	1707	C = O stretching unconjugated
5	1604	1630	1608	1610	1612	1616	Aromatic skeletal vibration +C = O stretching
6	1502	1520	1498	1502	1475	1514	Aromatic skeletal vibration
7	1432	-	1429	1427	1425	1412	C-H deformation (methyl and methylene
8	1408	1419	1398	1344	1385	1363	C-H in plane deformation with aromatic ring stretching
9	1315	1381	1313	1315	1363	1315	C-H cellulose +C-O of syringyl ring derivatives
10	1242	1306	1240	-	1313	-	C-O of guaiacyl ring
11	1225	1228	1223	1225	1228	1223	Siringyl ring +C-O stretch in lignin and xylan
12	1038	1039	1041	1039	1039	1082	C-O stretching in cellulose and hemicellulose
13	1024	998	1028	1018	1028	1026	C-H deformation vibrations of C-H bonds in aromatics rings
14	881	-	883	889	876	-	C-H deformation

We observed that biomass degradation differed according to tissue type (Figure S2). For the seed coat, xylem, stem, and leaf, the intensity of peak 12 (assigned as C–O stretching in cellulose and hemicellulose) and 14 (C–H deformation of amorphous cellulose) were unchanged at 200°C and 250°C, and a decrease was observed at 300°C and 350°C. For the kernel and bark, in contrast, the decrease in the intensity of peaks 12 and 14 started at temperatures lower than 200°C and 250°C, respectively. The decrease in these peaks is a hallmark of the reduction of cellulose and hemicellulose content by volatilization and condensation during char formation [40]. Thus, cellulose and hemicellulose degradation may start at 200°C in the kernel, 250°C in the bark, and 300°C in the seed coat, xylem, stem and leaf.

The tissue-specific degradation patterns were also observed for lignin and aromatics compounds (peaks 5, 6, 7, 8, 9, 10, and 11). For the seed coat and xylem, a rise in the temperature caused a gradual decrease in their peak intensities, although the peaks were present at 350°C. Therefore, it is possible that lignin and aromatic compounds gradually decomposed over large temperature ranges. However, for the bark, leaf, and stem, the same peaks increased in intensity with an increase in the temperature, particularly peak 5, which was assigned as an aromatic skeletal vibration, and peak 9, which was assigned as C–H cellulose +C–O of syringyl ring derivatives. Chen et al. reported that the presence of peaks 5 and 9 and peaks around 750 cm^−1^ represent calcium oxalate monohydrate [41]. This mineral widely occurs in plants, including the bark tissues and in the stone cells of phloem and leaf [42]. Calcium oxalate is known as thermal degradation over 500 degree C due to removal of carbon monoxide. It is possible to verify the heat resistance of calcium oxalate in the bark, leaf, and stem because the peak persists even at high temperatures, which is in agreement with the results of Sen et al. [40].

For the kernels, the pattern of the peaks assigned to aromatic compounds and lignins was different. Peaks 5–11 decreased as the temperature rose. It is possible that overlapping signals were derived from amino acids, as the kernel is rich in amino acids and poor in lignin [20]. Thus, it is possible that the degradation of amino acids started at 250°C and continued at higher temperatures.

### NMR analysis of HMWMs

**Figure 3:**
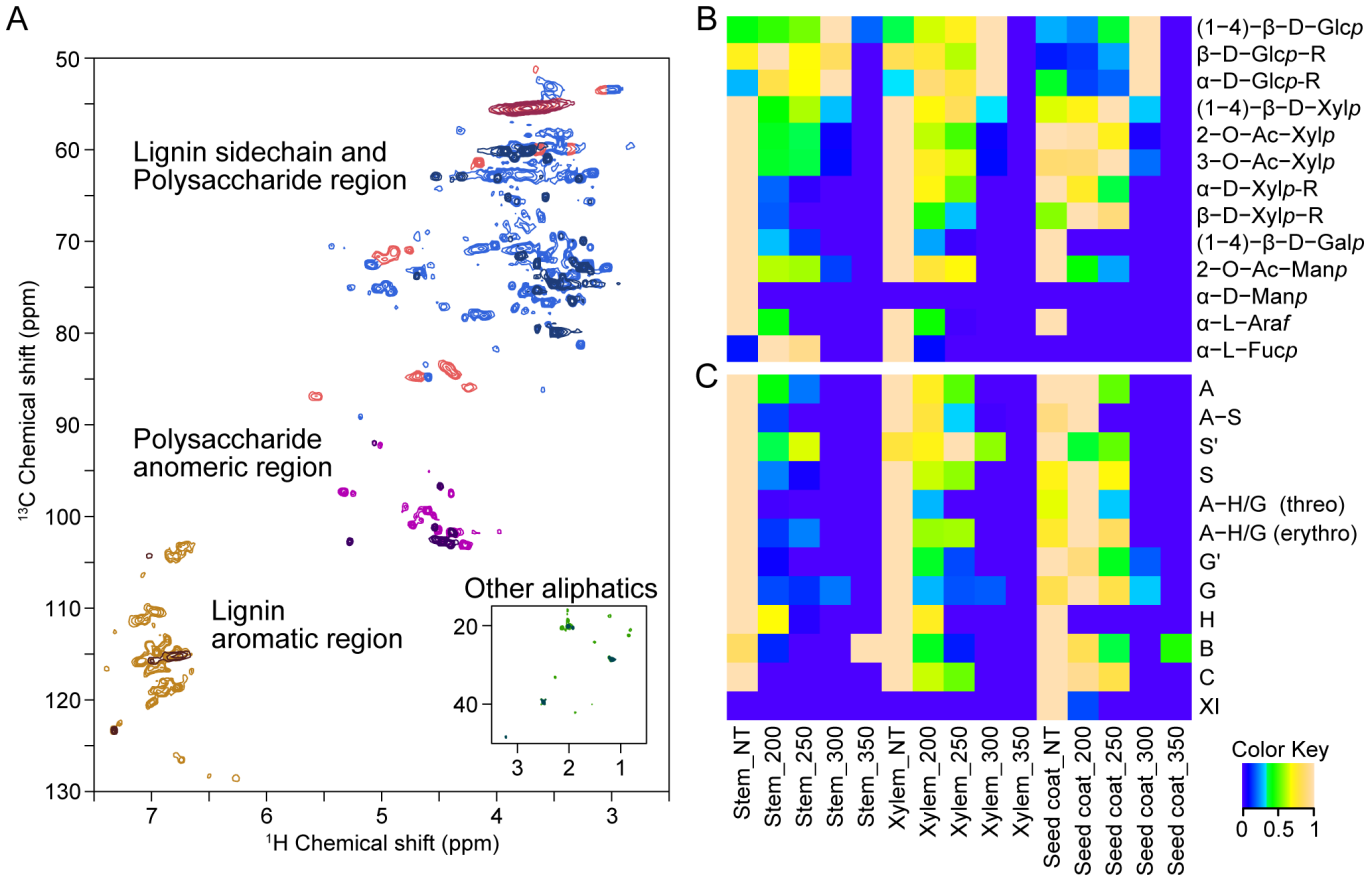
Comparison of non-treated and heat treated *Jatropha* biomass of 2D ^1^H–^13^C HSQC spectra. The 2D HSQC spectra of the seed coats heated at 200°C (lighter) and 350°C (darker) were divided into four regions as follows: lignin side-chain (red), polysaccharide (blue), polysaccharide anomeric (purple), lignin aromatic (brown), and other aliphatics (green) (A). Heat map of signals assigned to polysaccharides residues (B), lignin units and lignin substructure (C) detected using ^1^H–^13^C HSQC NMR analysis of the non-treated (NT) samples and stem, xylem, and the seed coat samples heated at 200°C (200), 250°C (250), 300°C (300), and 350°C (350).

The ^1^H–^13^C HSQC NMR spectra of the seed coats treated at 200°C (lighter) or 300°C (darker) were divided into four different regions as follows: lignin side-chain (red), polysaccharide (blue), polysaccharide anomeric (purple), lignin aromatic (brown), and other aliphatics (green) (Figure 3A). By observing both spectra, it is possible to verify the difference in signal intensities and numbers of signals. The degradation of HMWMs was analyzed in detail using the area of assigned signals from ^1^H–^13^C HSQC NMR to generate a heat map (Figure 3B, S3A).

We performed correlation analysis of the signals to determine the origin of certain polysaccharide residues. There was a high correlation among the signals assigned as (1–4)-β-D-glucopyranoside [(1–4)-β-D-Glc*p*], β-D-glucopyranoside reducing end (β-D-Glc*p*-R), and α-D-glucopyranoside reducing end (α-D-Glc*p*-R) residues (Figure S5). These residues are common components of cellulose and were likely derived from the cellulose in the samples.

Glucuronoxylan residues (1–4)-β-D-xylopyranoside [(1–4)-β-D-Xyl*p*], acetylated xylopyranoside (2-O-Ac-Xyl*p*, 3-O-Ac-Xyl*p*), α-D-xylopyranoside reducing end (α-D-Xyl*p*–R), and β-D-xylopyranoside reducing end (β-D-Xyl*p*-R) correlated highly with each other and were likely derived from glucuronoxylan (Figure S5). Other residues may have been derived from different polysaccharides, but there was not a high level of correlation.

#### Properties of cellulose

Figure 3B shows a heat map generated using the value of the area under the peak of each of the assigned polysaccharide residues and is scaled from 0 to 1. The heat map reveals an increase in the area values from the non-treated to 300°C-treated samples, but the signals were either undetectable or very low for all the samples heated to 350°C. The increase in the peak area may be explained by the cleavage of the glycoside linkages in the cellulose polymer that produced oligomers, which are more soluble in DMSO–pyridine and consequently increase the value. In all the samples except leaves, the highest intensity was generated by heating to 300°C, indicating that cellulose decomposition possibly started at low temperatures, increased significantly at 300°C, and was largely completed at 350°C. The values for leaves were the highest when heated to 250°C, decreased at 300°C, and were undetectable at 350°C (Figure S3B).

Pyrolysis of cellulose present in different types of biomass causes a primary fragmentation reaction associated with the cleavage of glycosidic bonds, which reduces the degree of polymerization and yields a tarry pyrolyzate containing levoglucosan, other anhydrosugars, oligosaccharides, and glucose decomposition products. It is followed by a secondary cracking reaction that produces volatiles. Fragmentation occurs at temperatures ranging from 200°C to approximately 300°C, and degradation occurs at higher temperatures [43], [44]. Our present FTIR and NMR results show that in most samples, cellulose was mainly fragmented to oligomers at temperatures up to 300°C, and degraded at higher temperatures (Figure 3B).

#### Properties of hemicelluloses

The products of thermal degradation of glucuronoxylan residues in the xylem, seed coat, bark, and stem showed a very similar pattern. Both end groups (α-D-Xyl*p*-R and β-D-Xyl*p*-R) were no longer detected in (1–4)- β-D-Xyl*p* and acetylated xylopyranoside residues after treatment at lower temperatures (Figure 3B, S3B). The signal intensity of acetylated xylopyranoside decreased with increasing temperatures, and low levels were detected at 300°C. In contrast, the signal intensity of (1–4)-β-D-Xyl*p* residues increased at 250°C, followed by an abrupt decrease at 300°C. At 350°C the signals were no longer detectable. Thus, it is possible that glucuronoxylan degradation started at its end groups.

In leaves, α-D-Xyl*p*-R was not detected in the samples that were not heated. For the seed coats and xylem, the intensities of the peaks of acetylated xylopyranoside residues were low at 300°C and 250°C, and the intensities of peaks of β-D-Xyl*p*-R were undetectable at 350°C and 300°C (Figure S3B). Signals representing 2-O-Ac-Xylp, 3-O-Ac-Xylp, and α-D-Xylp were not detected in the kernel samples; therefore, it is possible that glucuronoxylan is not present or was present at low levels, and that the (1–4)-β-D-Xylp residues may have been derived from a different polysaccharide (Figure S3B).

The degradation patterns of the other residues heated to 200°C or 300°C were not well defined compared with cellulose (Figure 3, S3). This difference can be explained by the heterogeneous composition of hemicellulose, in contrast to cellulose, that can vary greatly within a given biomass species [20]. In general, hemicellulose degrades between 150°C–350°C [18], [45], in agreement with our results showing that different residues of hemicellulose decomposed mainly between 200°C–300°C.

#### Properties of lignin

Lignins are aromatic heteropolymers composed of *p*-hydroxyphenyl (H), guaiacyl (G), and syringyl (S) units. Guaiacyl and syringyl can be found with oxidation in α-ketones (G′, S′). The coupling of lignin units form the following substructures: β-O-4 (A), β-5 (B), β-β (C), 5-5/4-O-β (D), 5-O-4 (E), and β-1(F). In this study we detected A, B, and C. The substructure A depended on the side chain represented as A-H/G when coupled to H or G units and as A–S when coupled to the S unit.

A heat map of the lignin units and substructure signals is shown in Figure 3C. Amino acids signals are known to overlap the signals from lignin, and thus, it was not possible to verify the decomposition pattern in the kernel, leaf, and bark, all of which contain a relatively high content of amino acids. The analysis of the heat map revealed that different lignin units degraded at discrete temperatures, depending on the sample. In the seed coats, H was no longer detected at 200°C; cinnamyl alcohol XI at 250°C; S and S′ at 300°C; and G and G′ at 350°C. In stems, G′ was no longer detected at 250°C; H, S, and S′ at 300°C; and G at 350. In xylem, H was no longer detected at 250°C; G′ and S at 300°C; and G and S′ at 350°C (Figure 3C).

Lignin decomposes over a broad temperature range, because its various oxygen functional groups have different thermal stabilities, and therefore their scission occurs at different temperatures. Further, the composition and structure of the lignin complex varies according to biomass type, reaction temperature, heating rate, and the degradation pattern [46], [47]. These properties can explain the difference in the decomposition pattern observed here.

Compared with the units, the substructures degraded at lower temperatures. In the seed coat samples, the substructures A–H/G (threo) and A–H/G (erythro) degraded at 300°C, G at 350°C, A–S at 200°C, and S at 250°C (Figure 3C). Similar results were observed for the other samples. Therefore, the thermal decomposition of lignin started with cleavage of the linkage followed by the degradation of the units.

The degradation of G and S, S and A–S occurred at lower temperatures compared with G and A–G/H (Figure 3C). The “A” between syringyl units is easier to cleave than that between guaiacyl units; therefore, S degradation occurred at an earlier stage of heating than G degradation [47]. Melkior et al. also demonstrated that during thermal decomposition, S moieties are decomposed to G by demethoxylation [48].

### NMR analysis of LMWMs

#### Water soluble (polar) LMWMs

**Figure 4:**
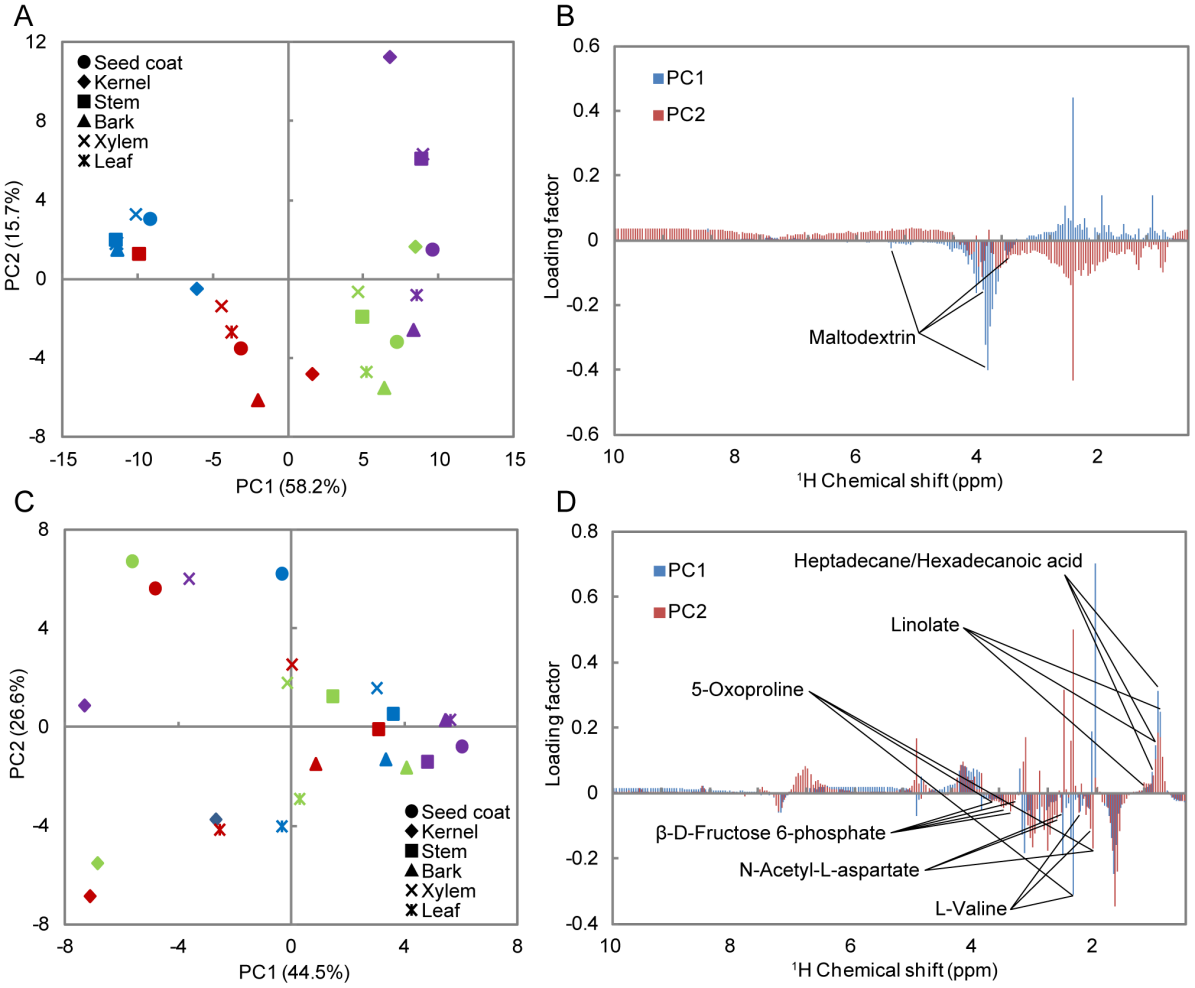
^1^H-NMR peak intensities of LMWMs analyzed using PCA. Upper PCA score plot (A) and loading plot (B) of water-soluble LMWMs. Lower PCA score plot (C) and loading plot (D) of methanol-soluble LMWMs. The samples treated at 200°C (blue), 250°C (red), 300°C (green), and 350°C (purple).

From the spectra of products generated by heated stems, it was possible to determine the predominant presence of maltodextrin signals at 200°C and particularly at 250°C. Cellobiose and succinate were present at relatively low levels. The intensity of maltodextrin signals increased at 250°C compared with 200°C, and signals were no longer detected at 300°C and 350°C (Figure S4A). The signals corresponding to maltodextrin in seeds were higher at 200°C, lower at 250°C, and undetectable at 300°C and 350°C (Figure S4B). From the score and loading plots of principal component analysis (PCA), all the samples treated at 200°C and stems treated at 250°C showed high intensity maltodextrin signals, because maltodextrin contributes to PC1 in the negative direction, and these plots are grouped at the negative side of PC1 in the score plot (Figure 4A, B). The plots for 300°C and 350°C are grouped along the positive side of PC1, indicating the absence of maltodextrin signals Thus, signals from maltodextrin were detected at 200°C and 250°C and no longer detected at 300°C and 350°C.

Maltodextrin (C_6_H_10_O_5_)nH_2_O_n_ is a polymer of saccharides that consists of glucose units primarily linked by α-1,4 glucosidic bonds and is derived from the hydrolysis of α-1,4 glucosidic bonds in starch. Thus, the presence of maltodextrin at 200°C and 250°C may indicate the decomposition of starch to low molecular weight molecules by the cleavage of the α-1,4 glucosidic bond. Previous high-resolution magic angle spinning NMR studies of intact *Jatropha* tissues indicated detection of sucrose signals [49]. However, this technique is not so adequate for the detection of immobile macromolecules, such as starch. Because the *Jatropha* stem possesses photosynthetic tissues on its green bark, the origin of starch may be attributed to these photosynthetic products. The starch in stems mainly decomposed to maltodextrin at 250°C, and maltodextrin and starch degraded at 300°C and 350°C, respectively. In the other samples, decomposition of starch mainly occured at 200°C, and starch and maltodextrin degraded at 300°C and 350°C. This result is in agreement with that of Liu et al. (2008), showing that starch is mostly degraded at 300°C when heated at 5°C/min, which is the same rate used in our study [50].

#### Methanol soluble (semipolar) LMWMs

We assigned the signals from metabolites generated by the products of fatty acid metabolism as heptadecane, tetracosanoic acid, hexadecanoic acid, and linoleic acid; sugars as β-D-fructose 6-phosphate; amino acids and compounds derived from amino acid metabolism as L-valine, N-acetyl-L-aspartate, and 5-oxoproline (Figure 4C). From PCA we identified three groups with different degradation patterns as follows: 1) stem, bark, and xylem; 2) seed coat, and 3) kernel and leaf. The ^1^H-NMR spectra and PCA for the bark, xylem, and stem were similar even at different temperatures. In these cases, the signals detected corresponded predominantly to fatty acids, which are common pyrolysis products that are generated by different biomass pyrolysis processes [51] (Figure 4C, D, S4C, D). The signal intensities of seed coats were compared with the other samples and therefore clustered separately (data not shown).

The high level of amino acids present in the kernels and leaves may have contributed to the different pattern of degradation, because L-valine, N-acetyl-L-aspartate, and 5- oxoproline contribute to the negative direction of PC1 and PC2 (Figure 4A). The spectra of the kernels (Figure S4D) and leaves (data not shown) show higher signal intensities for amino acids that were derived from the kernels at 250°C and from the leaves at 200°C. Therefore, peptides and amino acids may be degraded to LMWMs at 200°C and 250°C; with a higher decomposition rate at 250°C for the kernels and 200°C for leaves. LMWMs degraded at 300°C and 350°C. These results are in agreement with those of other studies that analyzed the thermal decomposition of amino acid using TG methods. In these studies, different types of amino acids were responsible for the highest weight-loss rate at approximately 300°C, which is the same temperature reported to decrease their signal intensities [52], [53], [54].

N-acetyl-L-aspartate is not present in plant tissue and is abundantly present in animal brain tissue. Therefore, it has been the focus of numerous studies on its roles in the nervous system. We identified this molecule using SpinAssign, and the four signals that were detected had the following chemical shifts: δC 39.469, δH 2.825, δC 52.631, δH 4.6304, δC 24.507, δH2.018, δC 39.156, and δH 2.757. The corresponding chemical shifts from the database were as follows: δC 39.408, δH 2.813, δC 52.55, δH 4.655, δC 24.214, δH 1.994, δC 39.408, and δH 2.785 ppm. It is therefore possible that this compound was generated by pyrolysis, but this must be verified using specific analyses.

### Decomposition of phorbol ester

**Figure 5:**
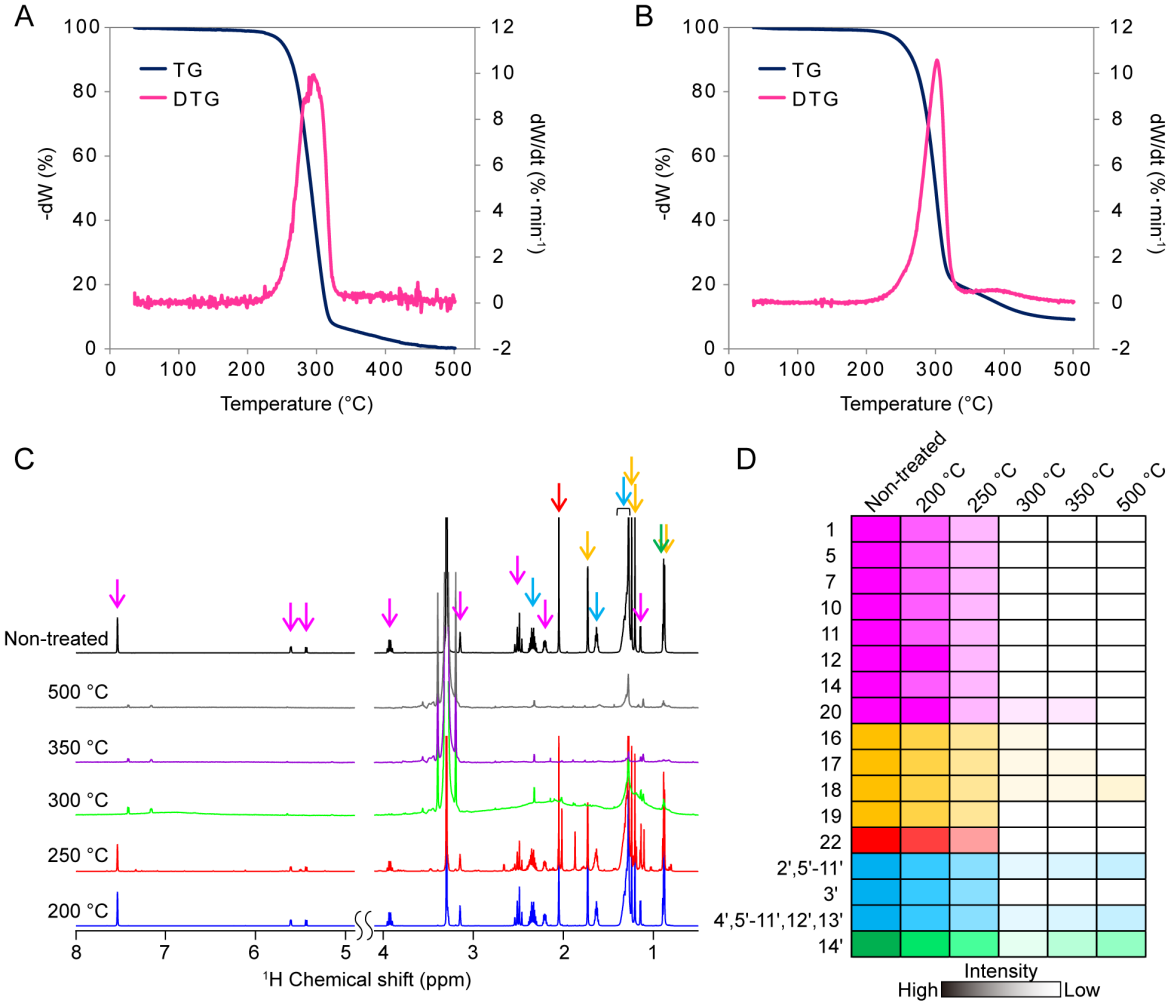
Analysis of the thermolysis process of the phorbol ester. Thermogravimetric–Differential Thermal Analysis of phorbol 12-myristate 13-acetate (A) and phorbol 12,13-dibutyrate (B). Stacked plots of ^1^H-NMR spectra heated phorbol 12-myristate 13-acetate at 200°C, 250°C, 300°C, 350°C, and 500°C (C), and changes in corresponding signal intensities against torrefication temperature (D). The coloring highlighted arrows on each signals in (C) are corresponded to same color in (D). Colors were classified as follows; aromatic (magenta), methyl (yellow), acetyl (red), lipid chain (cyan) and methyl derived from lipid chain (green).

The optimum temperature for torrefaction of phorbol 12-myristate 13-acetate and its di-butyrate form was first determined using thermogravimetric analysis at a heating rate of 5°C/min from 24°C to 500°C. Differential TG peaks were first detected in the samples heated at 250°C (5% weight decrease), peaked at 296°C (57% weight decrease), and decomposed at 350°C (94% weight decrease) (Figure 5A). We noted that a similar toxic compound, phorbol 12-myristate di-butyrate, exhibited a very similar thermal degradation property (Figure 5B). These torrefied samples (heated to 200°C, 250°C, 300°C, 350°C, and 500°C) were analyzed using ^1^H-NMR (Figure 5C), and the changes in relative signal intensities are shown in Figure 5D. Some aromatics and ester signals decreased from 250°C, suggesting that major moieties decomposed at lower temperature than side-chain aliphatics. Thus, this phorbol ester degraded at temperatures just below 300°C (Figure S6), indicating the suitability of these temperatures to detoxify biorefinary-stock and fertilizers.

#### FTIR and NMR results

The NMR and FTIR results suggested that the decrease in the intensity of peaks 12 to 14 between 300°C and 350°C was caused by the degradation of cellulose. The decrease between 250°C and 300°C was caused by the degradation of glucuronoxylan and between 200°C and 300°C by starch degradation. Because starch is composed of glucose residues as is cellulose, its degradation may have contributed to peak 12.

The decrease in peak 13 between 250°C and 300°C was more accentuated in the bark and kernel samples, which contain relatively lower levels of hemicellulose and cellulose [15]. Although the contribution of starch to peak 13 may be higher in these samples, cellulose residues degraded at 350°C, and xylopyranose-based residues (2-O-Ac-Xyl*p*, 3-O-Ac-Xyl*p*, α-D-Xyl*p*-R, β-D-Xyl*p*-R, (1–4)- β-D-Xylp) degraded at temperatures higher than 250°C, which may characterize the degradation of starch.

## Conclusions

Torrefaction of *Jatropha* tissues decomposed cellulose to oligosaccharides at increasing rates from 200°C to 300°C, and at 350°C cellulose and oligosaccharides derived from cellulose degraded. Each tissue yielded similar results. However, the decomposition of hemicellulose differed among the samples and was likely caused by the differences in the structures and compositions of hemicellulose samples. The analysis of LMWMs showed that starch decomposed to maltodextrin at 200°C and 250°C and degraded at 300°C and 350°C. Fatty acids that are commonly found in tar generated by torrefied biomass were present in all the samples heated to each temperature. Lignin decomposes over a wide temperature range. Lignin degrades in the order H, S, G. The substructures A–S, AH/G largely degraded at 250°C and 300°C, respectively, and their subunits degraded at 350°C. Amino acids and peptides (LMWMs) decomposed at 200°C and 250°C and degraded at higher temperatures. Phorbol ester degraded at temperatures just below 300°C. In most cases, heating biomass at 200°C had little effect, heating at 250°C produced smaller molecules, and complete degradation occurred at 300°C and 350°C.

## Supporting Information

Figure S1 **Thermogravimetric–Differential Thermal Analysis of** ***Jatropha*** **tissues.** The temperature was risen from 45°C to 500°C with a heating rate of 5°C/min.
(TIF)

Figure S2 **The FTIR spectra of** ***Jatropha*** **tissues.** Assignment of peaks in the FTIR spectrum of non-treated stem (A). The FTIR spectra of each tissues treated by different conditions; non-treated (NT) and treated at 200°C (200), 250°C (250), 300°C (300), and 350°C (350); Seed coat (B), Bark (C), Xylem (D), Leaf (E), Kernel (F), Stem (G).
(TIF)

Figure S3 **Clustered heat map by signal intensities from ^1^H–^13^C HSQC NMR analysis of all tissues.** The heat map of all signals intensities (A). The heat map was divided in signals derivate from aliphatic and anomeric regions, protein, aliphatic and aromatic regions and others. The heat map of signals assigned to polysaccharides residues (B). The samples heated at 200°C (200), 250°C (250), 300°C (300), 350°C (350), and non-treated (NT) for the seed coat, bark, xylem, leaf, kernel, and stem.
(TIF)

Figure S4 **The ^1^H-NMR spectra of LMWMs.** Spectra of water-soluble LMWMs in stems (A) and seed coat (B) treated at 200°C, 250°C, 300°C, and 350°C indicating maltodextrin, cellobiose, and succinate signals. Spectra of methanol-soluble LMWMs in bark (C) and kernel (D) samples treated 200°C, 250°C, 300°C, and 350°C showing heptadecane, tetracosanoic acid, β-D-fructose 6-phosphate, hexadecanoic acid, L-valine, linoleic acid, N-acetyl-L asparate, and 5-oxoproline.
(TIF)

Figure S5 **A correlation map from ^1^H–^13^C HSQC signal.** Correlation of signals detected using ^1^H–^13^C HSQC NMR analysis under each condition (bottom left) is divided into the regions of the NMR spectra and assigned signals. Amplification of the correlation map of assigned signals is denoted in the upper right.
(TIF)

Figure S6 **The ^1^H-NMR spectra of phorbol ester.** Chemical structure of phorbol 12-myristate 13-acetate and its thermal degradation profile showing in ^1^H-NMR stacked plots.
(TIF)
