# Supplementary figures and images for: Chemical Profiling of Jatropha Tissues under Different Torrefaction Conditions: Application to Biomass Waste Recovery
Source: PLoS One. 2014 Sep 5;9(9):e106893. doi: 10.1371/journal.pone.0106893 (PMC4156417; doi:10.1371/journal.pone.0106893)

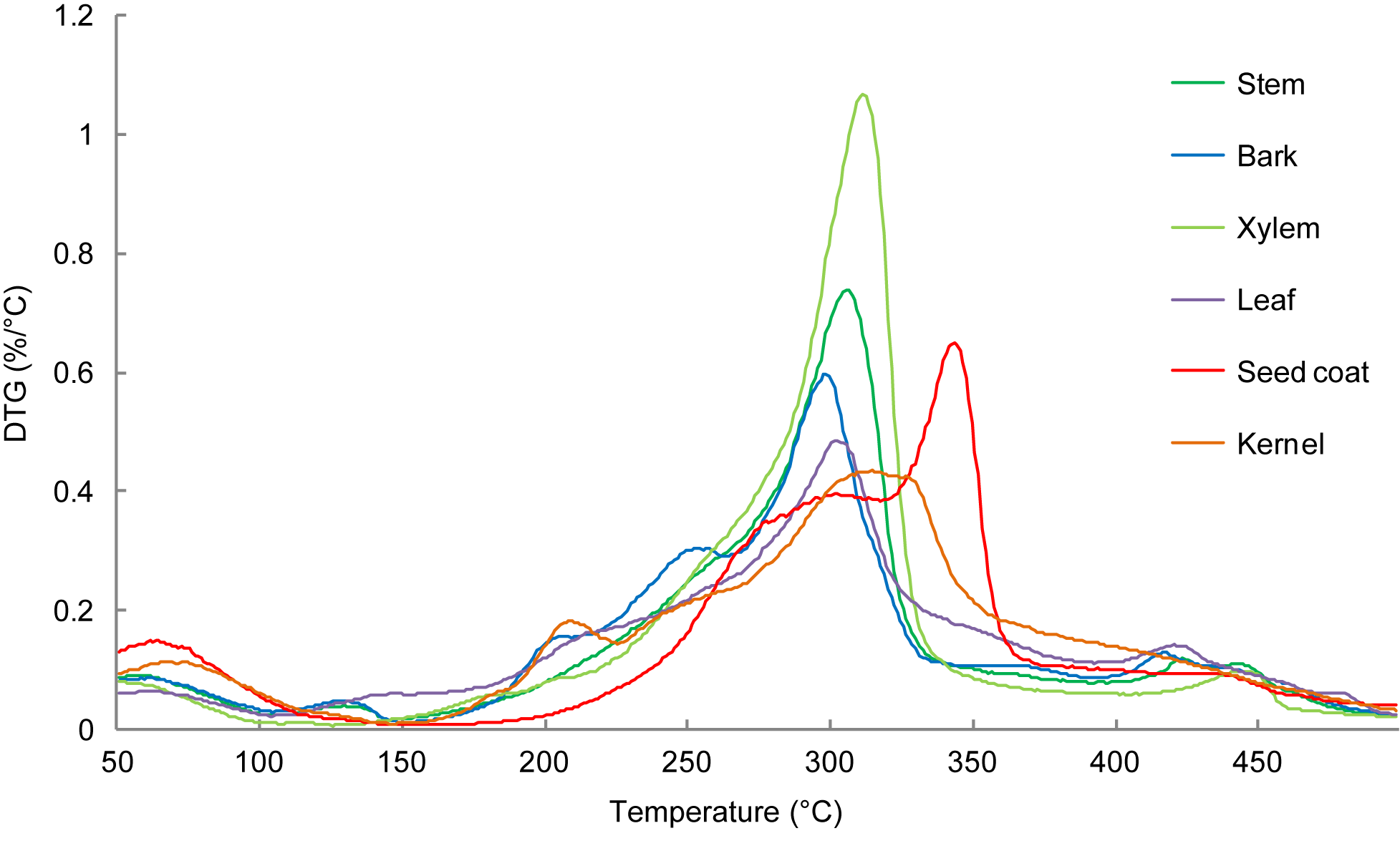

Supplement: Figure S1 — Thermogravimetric–Differential Thermal Analysis of Jatropha tissues. The temperature was risen from 45°C to 500°C with a heating rate of 5°C/min. (TIF) [file pone.0106893.s001.tif]

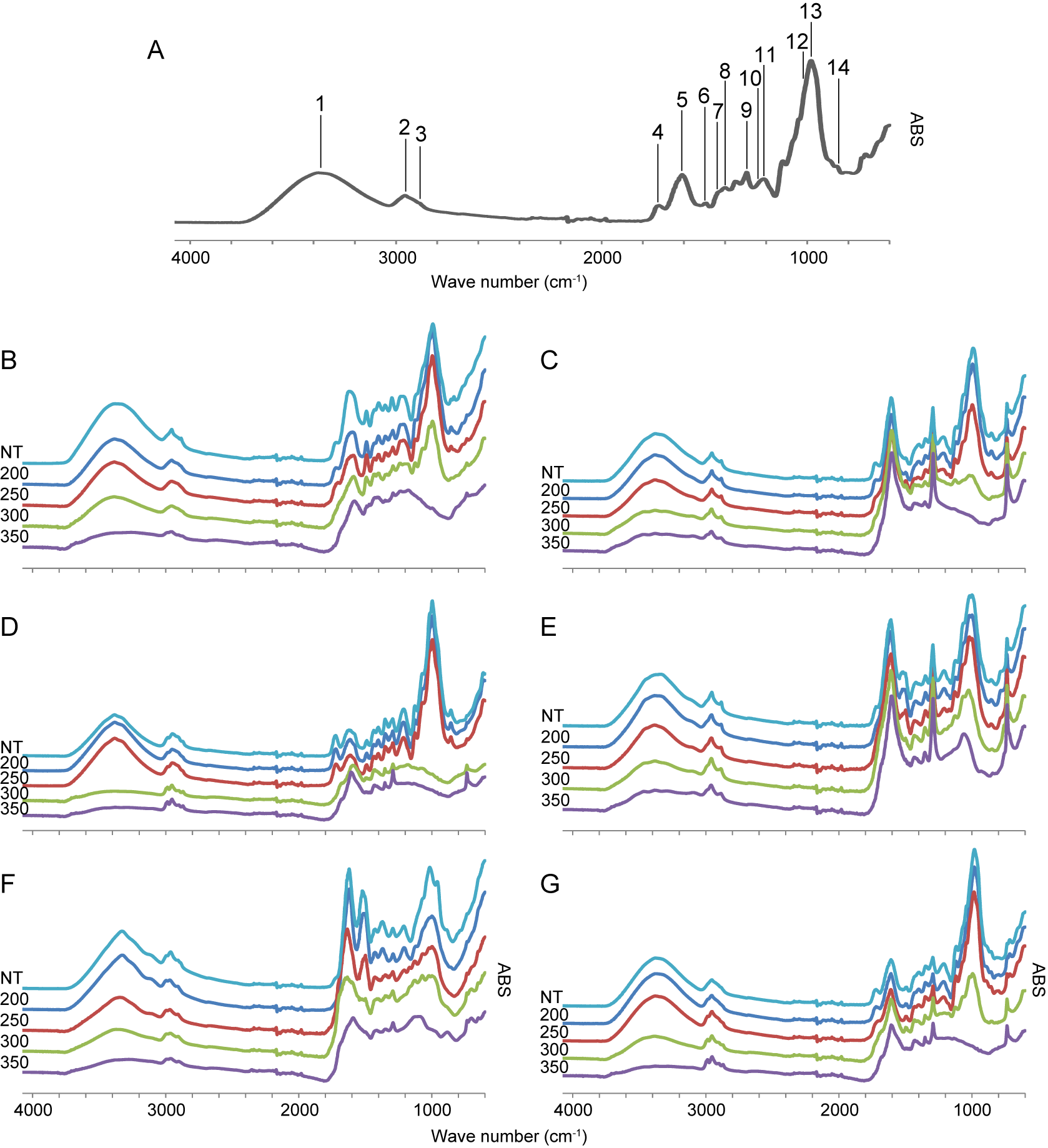

Supplement: Figure S2 — The FTIR spectra of Jatropha tissues. Assignment of peaks in the FTIR spectrum of non-treated stem (A). The FTIR spectra of each tissues treated by different conditions; non-treated (NT) and treated at 200°C (200), 250°C (250), 300°C (300), and 350°C (350); Seed coat (B), Bark (C), Xylem (D), Leaf (E), Kernel (F), Stem (G). (TIF) [file pone.0106893.s002.tif]

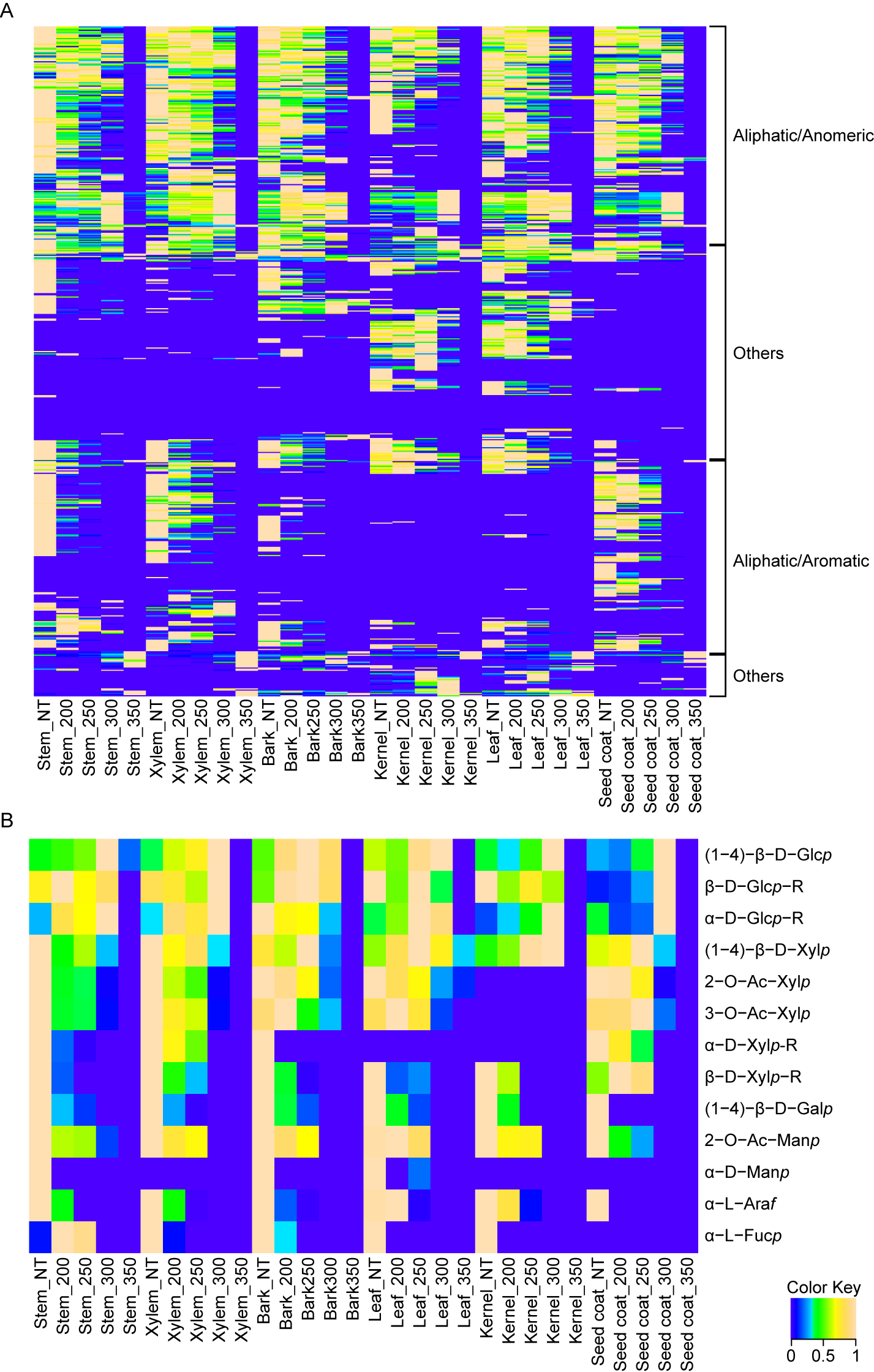

Supplement: Figure S3 — Clustered heat map by signal intensities from 1H–13C HSQC NMR analysis of all tissues. The heat map of all signals intensities (A). The heat map was divided in signals derivate from aliphatic and anomeric regions, protein, aliphatic and aromatic regions and others. The heat map of signals assigned to polysaccharides residues (B). The samples heated at 200°C (200), 250°C (250), 300°C (300), 350°C (350), and non-treated (NT) for the seed coat, bark, xylem, leaf, kernel, and stem. (TIF) [file pone.0106893.s003.tif]

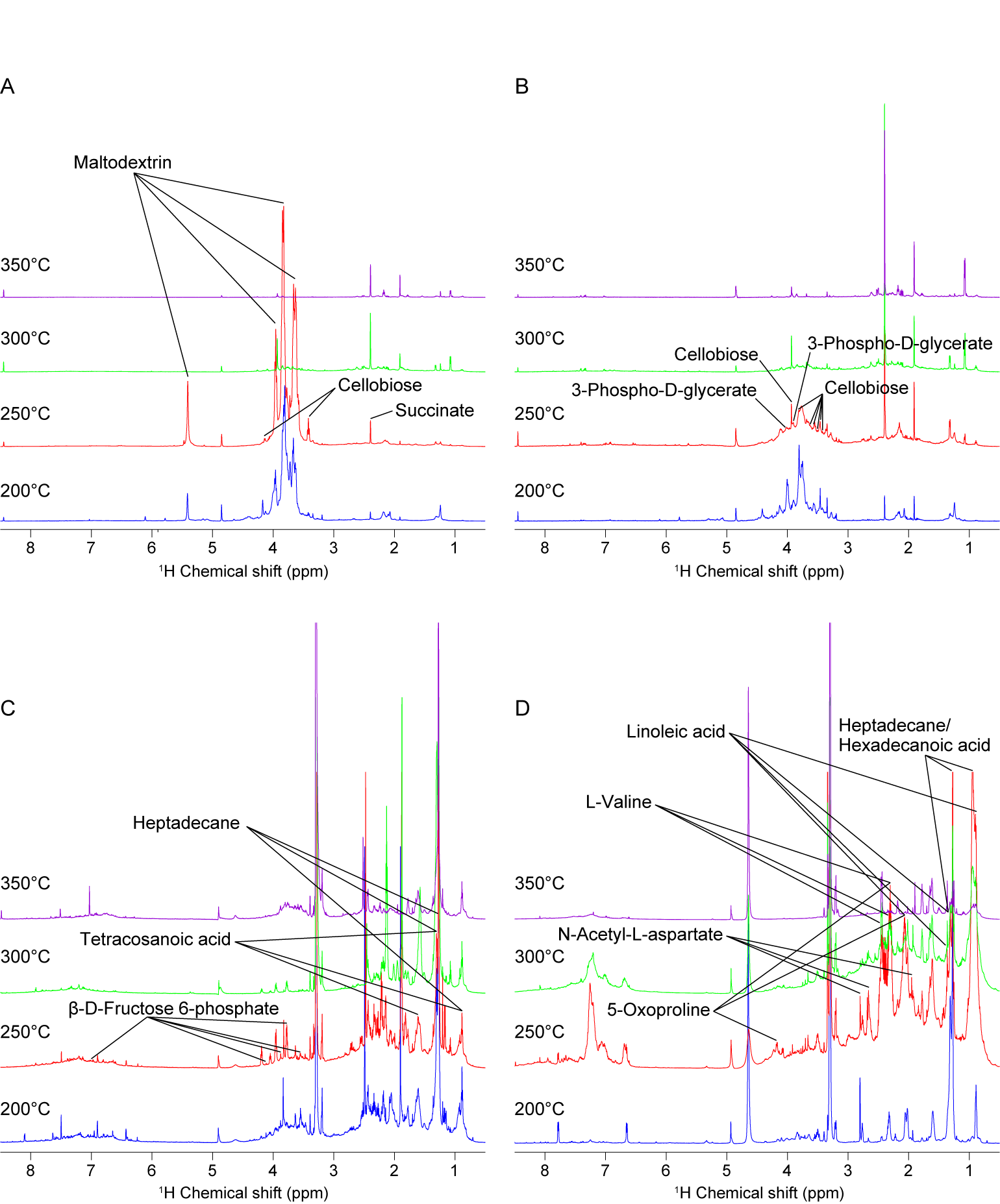

Supplement: Figure S4 — The 1H-NMR spectra of LMWMs. Spectra of water-soluble LMWMs in stems (A) and seed coat (B) treated at 200°C, 250°C, 300°C, and 350°C indicating maltodextrin, cellobiose, and succinate signals. Spectra of methanol-soluble LMWMs in bark (C) and kernel (D) samples treated 200°C, 250°C, 300°C, and 350°C showing heptadecane, tetracosanoic acid, β-D-fructose 6-phosphate, hexadecanoic acid, L-valine, linoleic acid, N-acetyl-L asparate, and 5-oxoproline. (TIF) [file pone.0106893.s004.tif]

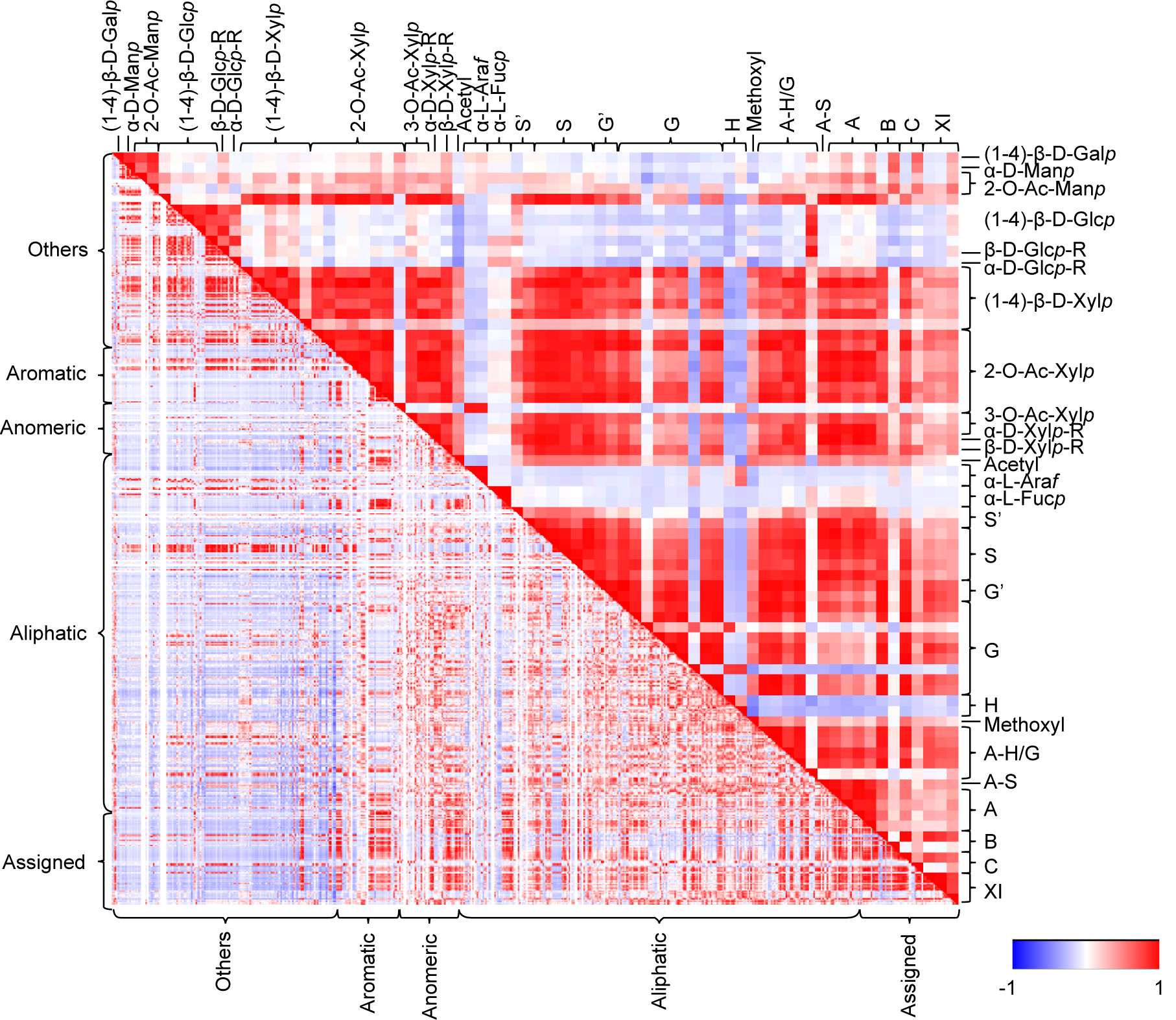

Supplement: Figure S5 — A correlation map from 1H–13C HSQC signal. Correlation of signals detected using 1H–13C HSQC NMR analysis under each condition (bottom left) is divided into the regions of the NMR spectra and assigned signals. Amplification of the correlation map of assigned signals is denoted in the upper right. (TIF) [file pone.0106893.s005.tif]

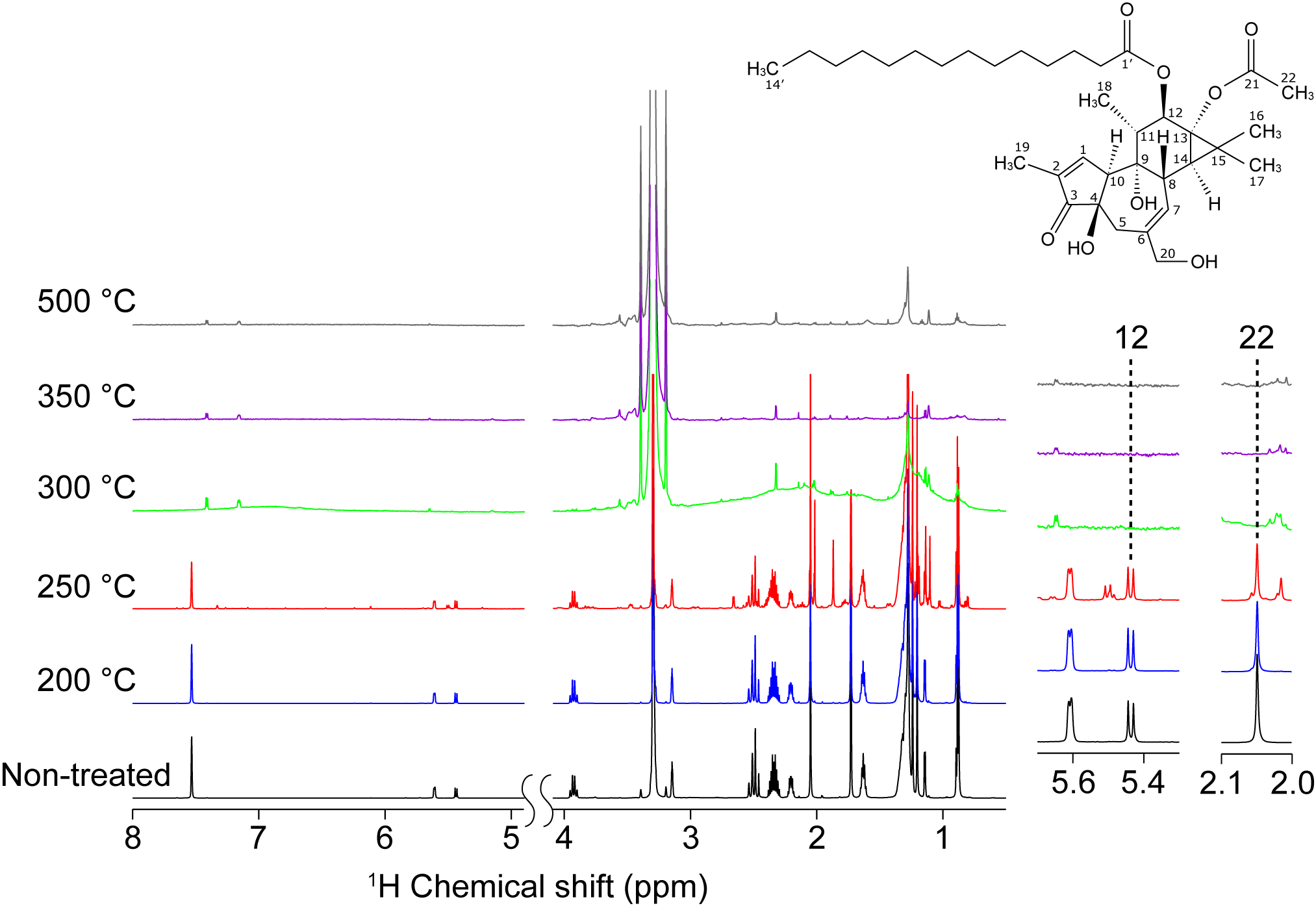

Supplement: Figure S6 — The 1H-NMR spectra of phorbol ester. Chemical structure of phorbol 12-myristate 13-acetate and its thermal degradation profile showing in 1H-NMR stacked plots. (TIF) [file pone.0106893.s006.tif]
